# Survival of Prosthodontic Restorations Luted with Resin-Based versus Composite-Based Cements: Retrospective Cohort Study

**DOI:** 10.3390/ma15010312

**Published:** 2022-01-02

**Authors:** Ján Staněk, Abanoub Riad, Adam Le, Matěj Bernát, Milad Hammal, Basel Azar

**Affiliations:** 1 Department of Prosthetic Dentistry, Faculty of Medicine and Dentistry, Palacký University Olomouc, 775 15 Olomouc, Czech Republic; miladhammal@gmail.com (M.H.); baselazar1@hotmail.com (B.A.); 2 Department of Public Health, Faculty of Medicine, Masaryk University, 625 00 Brno, Czech Republic; abanoub.riad@med.muni.cz; 3 Private Dental Practice, 413 01 Vědomice, Czech Republic; leadam97@gmail.com; 4 Private Dental Practice, 760 01 Zlín, Czech Republic; bernat.matej@seznam.cz

**Keywords:** cohort studies, complications, dental cements, inlay, onlay, overlay, resin cements, survival rate

## Abstract

The purpose of this study was to evaluate clinical performance, survival, and complications of indirect composite inlays, onlays, and overlays on posterior teeth. Digital records of 282 patients treated between 2014 and 2018 were accessed and analyzed retrospectively. The included patients received 469 composite restorations luted with seven different resin-based types of cement, i.e., Filtek Ultimate Flow, Enamel Plus, Relyx Ultimate, Harvard Premium Flow, Relyx Unicem, Filtek Bulk Fill Flowable, and Filtek Ultimate. The restorations had been clinically and radiographically evaluated annually. The mechanical and clinical complications, e.g., debonding, fracture, and secondary caries, were evaluated and recorded. The examined restorations exhibited a high survival rate (84.9%), and failure was found in only 71 cases. Fracture was the most common cause (n = 36), followed by prosthetic work release (n = 19) and secondary caries (n = 16). There was a statistically significant difference between failure and cement material (Sig. < 0.001); the composite-based cements (87.2%) had a high survival rate compared to the resin-based cement (72.7%). Similarly, the cements with high viscosity (90.2%) had significantly higher survival rates than the low-viscosity cements (78.9%). Moreover, onlays showed higher longevity compared to overlays (Sig. = 0.007), and patients aged under 55 years showed less complications (Sig. = 0.036). Indirect composite restoration was a successful solution to tooth structure loss. The material of the cementation is an important part of the success. Higher survival rate was found in our study when the fixation materials with high viscosity were used, thus suggesting using these materials with indirect restorations. Composite-based cements had significantly higher survival rate than resin-based cements.

## 1. Introduction

In clinical practice, hard dental tissue loss caused by decay, erosive or abrasive wear, trauma, or a combination often exceeds direct restoration limits. According to Pjetursson et al. 2008, the preparation for conventional crown luted with conventional cement leads to additional loss of dental tissues [1]. In a recent systematic review, Angeletaki et al. 2016 concluded that the indirect approach is recommended when the cusp is lost or the defect exceeds the occlusal third [2].

Indirect composite restorations offer better mechanical properties than direct composite restorations, such as higher wear resistance and lower polymerization shrinkage [3]. The indirect process of fabrication offers better access for creating natural occlusal and interproximal design. While the degree of conversion influences fracture resistance and wear resistance, the time for proper degree of conversion is not patient-dependent during indirect fabrication [4,5].

Contemporary computer-aided design and manufacturing (CAD/CAM) materials offer the clinician a huge variety of different materials with different optical and mechanical properties able to fulfill almost any type of clinical situation [6,7]. Given the rapid development of CAD/CAM technology, single visit indirect restoration has emerged as a treatment modality with comparably reduced treatment time; however, it still brings additional costs and it is still more time consuming compared to the direct restoration [8,9,10].

In general, indirect partial restoration can be fabricated from ceramic, composite, or hybrid materials [11]. Composite can be easily modified and repaired, in contrast with ceramic restorations that offer better wear resistance [12,13], even though it had been recently reported that ceramic onlays experience more marginal fractures [14]. The additional tissue loss can be avoided by using minimally invasive prosthetic designs, such as overlays, onlays, and inlays [15,16,17]. Adhesive luting materials have been widely used because they are a more conservative approach that preserves larger amounts of dental tissues [18,19,20]. However, the adhesive cementation is more conservative compared with retentive preparation; it has several challenges, e.g., moisture control, technique sensitivity for surface pretreatment of tooth and restoration, material selection, and proper manipulation, which are crucial for restoration longevity [21,22,23,24,25].

The dental tissues are one of the surfaces for adhesion. Given that enamel is composed of up to 96% of inorganic substances, the adhesion of enamel can be highly successful. On the other side, dentin is a wet tissue containing tubules composed of up to 30% of the organic matrix, where the diameter of the tubules and humidity of the dentin increases gradually in proximity to the dental pulp [26]. With regard to composite restorations, the degree of polymerization under the restoration is crucial and can be affected by opacity and thickness of the restorative material, which can reduce light translucency and proper polymerization [27]. The luting cements are used to prevent restoration release, microleakage, decay, and esthetic defects [2]. The proper seal by luting material is required; therefore, high tensile and compressive strength and low solubility are fundamental characteristics. Moreover, the high modulus of elasticity offers retention during functioning of the restoration [28,29,30].

The resin-based luting cements can be classified according to the polymerization technique, i.e., light-cured, dual-cured, and chemically cured, or they can be classified according to tooth pretreatment and adhesive scheme, i.e., total etch resin cements, self-etch resin cements, and self-adhesive resin cements [31,32]. Total etch resin cements require application of phosphoric acid on dentin and enamel; as a result, the smear layer is removed and dentinal tubules are exposed. This way of luting leads to the highest bond strength; however, multiple steps that are necessary can jeopardize the success [33,34]. Zidan et al. 2003 found that the bonding strength on adhesively luted abutment with conicity of 24° was higher than the tooth with conicity of 6° luted with conventional cement [35]. Self-etch resin-based cements apply etching primer on dentin while the bonding strength to enamel is significantly weaker compared to the total etch [36]. Self-adhesive resin-based cements are used without tooth surface pretreatment, and they contain phosphoric acid to achieve sufficient adhesion to both enamel and dentin [37,38,39,40]. The proper setting of the restoration using light-cured cements can be achieved in a longer time for controlling the removal of excesses. Increasing the fixation material that is liquid increases the odds of bubble formation, and the preheated composites can create a marginal increase [41,42].

Light-cured cements offer higher color stability over the time compared to the dual or chemically cured cements [31]. Self-adhesive cements show better bondability to the dentin than to the enamel. Bonding to enamel can be improved by selective etching and application of bonding agent on the enamel. However, the same procedure, when applied on the dentin, leads to a decrease in bonding strength [39].

Conventional composites can be used as luting cement. Flowable composite contains 37–53% of fillers that increase the flowability, and their high viscosity allows proper setting of the restoration while the shrinkage is larger compared to the conventional composites [43]. One the advantages of using flowable composites is creating the so-called “super dentin” and acid-resistant zone, which can prevent secondary caries [44,45]. Flowable composites had been suggested as alternatives to dual-cured resin cements when the restoration is thinner than 2 mm. There is a huge variability within the group of flowable composites according to radiopacity, flowability, filler content, modulus of elasticity, and flexural strength ranging from 66 to 145 MPa [46,47,48,49,50].

Preheating composites makes the placement of restoration easier, the conversion of the monomer higher, and the optical proprieties maintained [51,52]. However, preheating of the composites allows better adaptability and it leads to an increased shrinkage. The volume of the preheated composite layer is higher compared to the resin-based cements or flowable composites and the marginal gap can be increased [42,53,54,55]. Both the preheated composite and flowable composite can be used with the total-etch protocol. The self-etch protocol does not require removal of the smear layer, but it modifies the smear layer and incorporates it [56].

In light of the previous findings, this study was designed to study indirect composite restorations due to the lack of evidence-based recommendations for the most appropriate luting cement used with composite restorations. The overarching aim of this study was to describe the outcomes of composite restorations luted with different cement materials over five years of post-installation follow-up.

## 2. Materials and Methods

### 2.1. Design

This is a retrospective cohort study, for which the patients received indirect prosthetic restorations luted with different resin-based cements and they were checked annually. The study was designed, conducted, and reported according to the Strengthening the Reporting of Observational studies in Epidemiology (STROBE) guidelines for cohort studies [57] (Supplementary Materials Table S1).

### 2.2. Setting

The study protocol was reviewed and approved by the Ethics Committee of the Faculty of Medicine and Dentistry, Palacký University Olomouc Ref. No. 223/21. All the patients were recruited and treated at an academic specialty facility, the Department of Prosthodontics, Palacky University Hospital in Olomouc, the Czech Republic, between January 2014 and October 2018. Each patient was checked annually, and all the prosthetic restorations were fabricated by one dental laboratory affiliated with the university hospital. The clinicians who delivered the restorations followed the same protocol.

Preparation of the teeth was performed under local anesthesia according to conventional principles for adhesive onlay preparation. At least one cusp was covered and the convergence angle was around 10°, with the limitation of free-hand preparation. The margins were prepared as a butt joint. After the preparation polishing, the immediate dentin sealing was performed under rubber dam and a eugenol-free provisional filling was placed.

The composite onlays were fixed using the following protocol; after the initial try-in, the restoration was sandblasted on the inner surface with aluminum dioxide 25 μM and cleaned with an ultrasonic cleaner for 2 min and air dried, and a silane agent (Ultradent) was applied for one minute, then the rubber dam was placed [58,59]. The inner surface of the restoration was treated with the adhesive system without polymerization. The tooth surface was prepared in accordance with manufacture recommendation for luting material. Composite that was used to block undercuts or as a coronal seal was sandblasted with aluminum dioxide 25 μM, then rinsed for 40 s and air dried. The tooth surface was prepared according to cement type. The excesses were removed using a scalpel, micro brush, super floss, and were eventually polymerized for 20 s six times from different aspects of the tooth. Occlusion was checked and the fit was checked with radioisotope thermoelectric generator (RTG).

### 2.3. Sample

**Figure 1:**
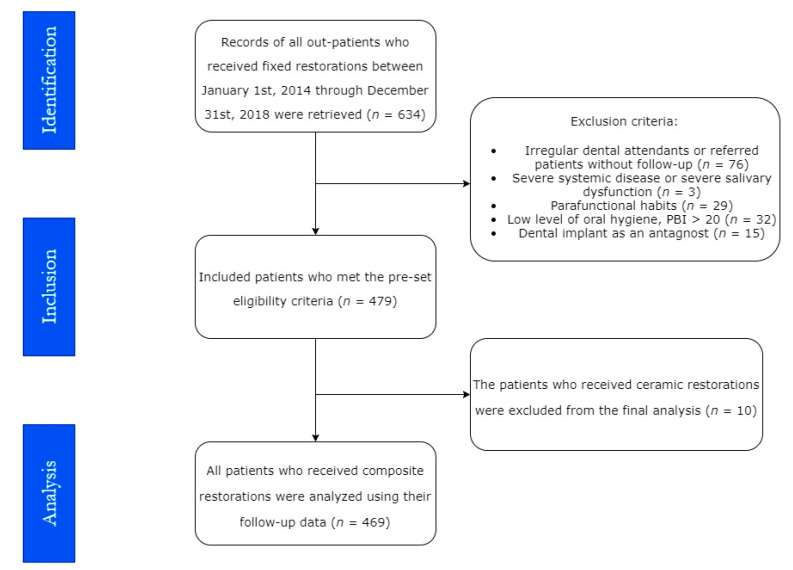
Flowchart of the study on composite restoration recipients, Palacky University Hospital, 2014–2018 (*n* = 469).

A total of 282 patients received 469 composite inlays, onlays, and overlays on either posterior vital or nonvital teeth following the manufacturers’ instructions and the guidelines for fixation in adhesive dentistry of Mante et al. 2013 [21]. The participants were included in this study according to the following eligibility criteria (Figure 1).

The inclusion criteria were (a) vital or nonvital premolars or molars (posterior teeth), (b) the prosthetic work should be either inlay, onlay, or overlay, (c) the recipient tooth should have at least one adjacent and one antagonist tooth, and (d) rubber dam use to ensure isolation.

The exclusion criteria were (a) irregular dental attendants or delegated patients with no follow-up data, that is the patients who started the study but they did not visit prosthodontists for follow-up; therefore, they were depicted as dropped out from the study (*n* = 76); (b) severe systemic diseases or severe salivary gland dysfunction (*n* = 3); (c) parafunctional habits, e.g., grinding and thumb-sucking (*n* = 29); (d) low level of oral hygiene indicated by a PBI score above 20 (*n* = 32); (e) tooth that does not require cuspal coverage; and (f) implant an antagonist (*n* = 15).

Epi Info ^TM^ version 7.2.4 (CDC. Atlanta, GA, USA, 2020) was used to compute the required sample size for this study. Following the assumptions of power test 80%, confidence level 95%, exposed-to-unexposed ratio 6:1, and outcome probability of exposed 79% and unexposed 55% based on previous literature, the required sample was 256 [60,61].

### 2.4. Outcome Measures

The primary outcomes of this study were prosthesis survival and success rate. At the annual checkups, the onlays were examined visually with mirror and probe, then the interdental space was examined with dental floss. Each restoration was examined for cracks, fracture, debonding, and marginal color changes. The patients were questioned for postoperative complaints. It was examined if there was need for restoration replacement. Bravo score and Charlie score were recorded.

### 2.5. Data Sources

Two independent investigators (A.L. and M.B.) extracted the patients’ data from the hospital database in December 2019. The electronic verification of patients’ cards was carried out. The year of onlay delivery was identified, then the annual follow-ups and other visits of the patient were checked. During the annual follow-up sessions, the indirect restorations were examined using the modified United States Public Health Service (USPHS) criteria for retention, color matching, marginal discoloration, marginal adaptation, secondary caries, surface texture, anatomic form, and postoperative sensitivity [62].

Annual checkups had been performed by prosthodontists. Bite-wings or periapical radiographs were taken according to indication of a restorative dentist. Radiographs were then evaluated during retrospective research for the presence of secondary caries by the investigators. This was combined with clinical examination and a decision about the presence of secondary caries was made.

### 2.6. Bias

To reduce the measurement bias, the investigators who checked the protocols in patient cards, were independent and did not work at the University Hospital (A.L. and M.B.); then they discussed the records with the senior investigators (J.S. and B.A.).

### 2.7. Analysis

All statistical tests were executed using the Statistical Package for the Social Sciences (SPSS) version 28 (SPSS Inc. Chicago, IL, USA, 2021) [63]. Primarily, descriptive statistics were carried out to describe the participant demographics and clinical characteristics, as well as their treatment outcomes, using frequencies (*n*), percentages (*%*), mean, and standard deviations (*µ* ± SD). Subsequently, regression analysis was performed for the significant risk factors of restorations failure, and time-to-event (Kaplan-Meier) analysis was executed to compare the restorations survival across age groups, cement materials, and cement viscosity. All inferential tests were conducted with the assumptions of confidence level 95% and significance level < 0.05.

## 3. Results

### 3.1. Sample Characteristics

**Figure 2:**
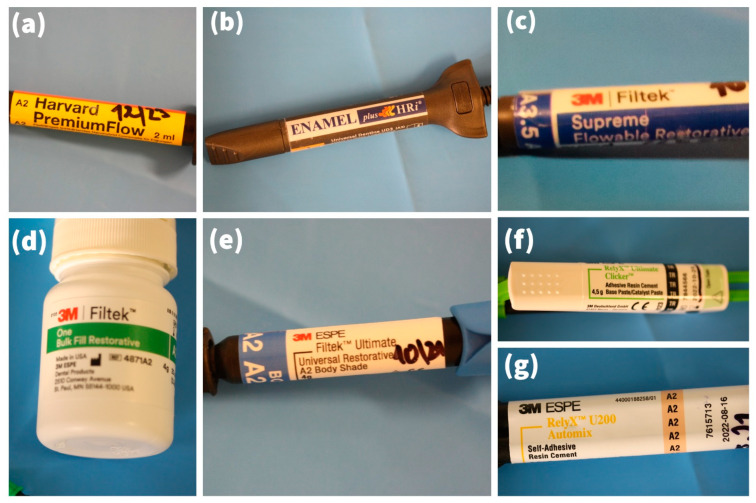
Luting cements that were used in this study: (**a**) Harvard Premium Flow; (**b**) Enamel Plus; (**c**) Filtek Ultimate Flow; (**d**) Filtek Bulk Fill Flowable; (**e**) Filtek Ultimate; (**f**) Relyx Ultimate; (**g**) Relyx Unicem, Palacky University Hospital, 2014–2018 (*n* = 469).

Out of the 469 included subjects, 271 (57.8%) were received by females, 354 (75.5%) by patients aged 55 years or below, and 237 (50.5%) in the upper jaw. Onlays (79.3%) were the most commonly placed composite restoration, followed by overlays (19.6%) and inlays (1.1%). Most restorations were luted with Enamel Plus (Micerium S.p.A., Genoa, Italy) (51%), followed by Harvard Premium Flow (Harvard Dental International GmbH, Hoppegarten, Germany) (19.8%), Relyx Ultimate (3M Company, Maplewood, MN, USA) (13.9%), and Filtek Ultimate Flow (3M Company, Maplewood, MN, USA) (10%) (Figure 2).

While the majority of used cements were composite-based (83.6%), almost half of them were with high viscosity (52.5%). The year 2016 had the highest share of composite restorations (36.7%), while the year 2018 had the lowest share (4.1%). Until 2019, 398 (84.9%) of the restorations survived and remained functional, while 36 (7.7%) broke down, 19 (4.1%) had leaked filling, and 16 (3.4%) manifested secondary caries underneath or around (Table 1).

### 3.2. Survival vs. Failure

**Figure 3:**
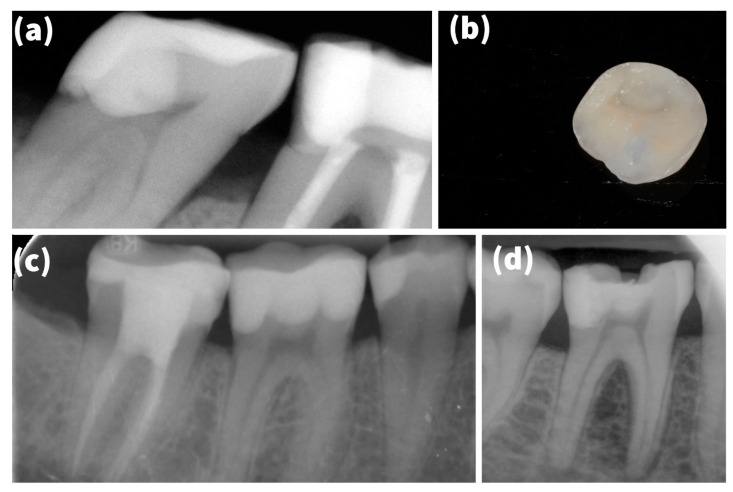
Outcome-related findings: (**a**) failed onlay on 47 because of secondary caries; (**b**) failed overlay after sandblasting in lab; (**c**) fitted onlay after two years; (**d**) fractured onlay; Palacky University Hospital, 2014–2018 (*n* = 469).

On comparing the survival versus failure rates across the potential risk factors, females (84.5%) had a similar survival rate to males (85.4%) (*χ^2^* = 0.065; Sig. = 0.799). The patients aged 55 years or below had a significantly (*χ*^2^ = 4.378; Sig. = 0.036) lower failure rate (13.3%) compared to the older patients (21.4%). However, the upper arch had a higher survival rate (87.3%) than the lower arch (82.3%); this difference was not statistically significant (*χ*^2^ = 2.356; Sig. = 0.125) (Figure 3).

Onlays exhibited a slightly and insignificantly (*χ*^2^ = 1.901; Sig. = 0.515) lower survival rate (83.9%) compared to overlays (88%). The restorations luted with composite-based cements had a significantly (*χ*^2^ = 10.558; Sig. < 0.001) higher survival rate (87.2%) than those luted with resin-based cements (72.7%). Similarly, the cements of high viscosity had a significantly (*χ*^2^ = 11.667; Sig. < 0.001) higher survival rate (90.2%) than those of low viscosity (78.9%) (Table 2).

### 3.3. Crude Longevity

The crude longevity is the number of years where the restoration remained functional, either until the end of follow-up in 2019 or until its failure. The mean longevity of all included subjects was 2.62 ± 1.08 years. There was no statistically significant difference across sexes (U = 25888; Sig. = 0.991), age groups (U = 17881; Sig. = 0.255), or arches (U = 25439.5; Sig. = 0.514).

On the other hand, onlays had significantly (U = 13633.5; Sig. = 0.007) higher longevity (2.66 ± 1.09) compared to overlays (2.43 ± 1.07). Similarly, the cements with high viscosity had significantly (U = 29849; Sig. = 0.011) higher longevity (2.74 ± 0.97) than those with low viscosity (2.48 ± 1.18). The resin-based cements (2.66 ± 1.46) and composite-based (2.61 ± 1.00) cements did not have statistically significant (U = 14999.5; Sig. = 0.475) difference in terms of crude longevity (Table 3).

### 3.4. Failure Risk Factors

On running binary logistic regression, gender, arch, and restoration type were adjusted to evaluate the impact of potential risk factors on the odds of restoration failure. The patients older than 55 years had 1.69 (CI 95%: 0.97–2.94) times of adjusted odds ratio (AOR) for restoration failure compared to their younger peers. The resin-based cements had an AOR of 2.90 (CI 95%: 1.59–5.29) and the cements with low viscosity had an AOR of 2.57 (CI 95%: 1.50–4.41) (Table 4).

### 3.5. Survival Analysis

To analyze the simple survival rates of composite restorations, Kaplan-Meier analysis was performed with a significance level of ≤ 0.05 (Table 5).

**Figure 4:**
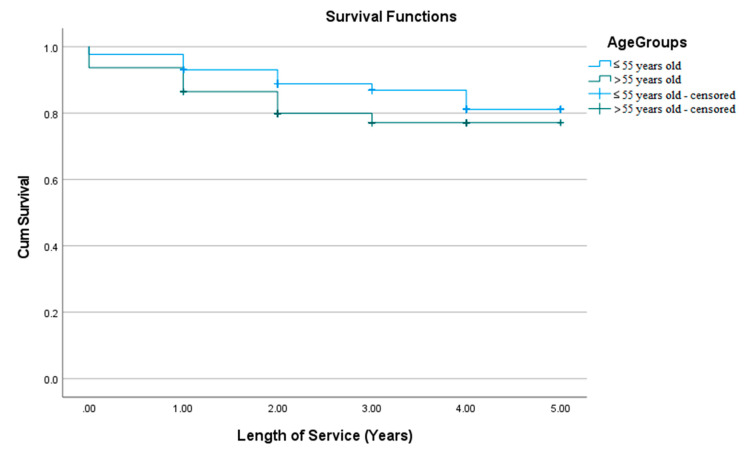
Kaplan-Meier survival curve of composite restorations stratified by age group, Palacky University Hospital, 2014–2018 (*n* = 469). CumSurvival = cumulative survival.

The younger age group had significantly (*χ*^2^ = 4.514; Sig. = 0.034) higher survival (4.48 ± 0.07) than the older age group (4.14 ± 0.16) (Figure 4).

**Figure 5:**
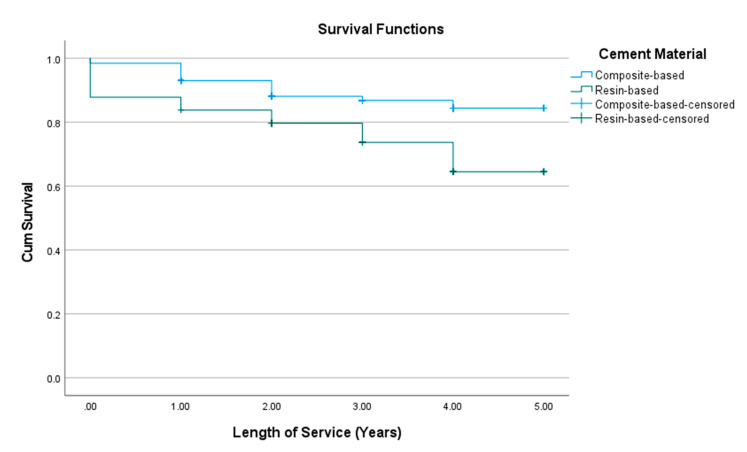
Kaplan-Meier survival curve of composite restorations stratified by cement material, Palacky University Hospital, 2014–2018 (*n* = 469). CumSurvival = cumulative survival.

The composite-based cements had significantly (*χ*^2^ = 10.310; Sig. = 0.001) higher survival (4.51 ± 0.07) than the resin-based cements (3.90 ± 0.21) (Figure 5).

**Figure 6:**
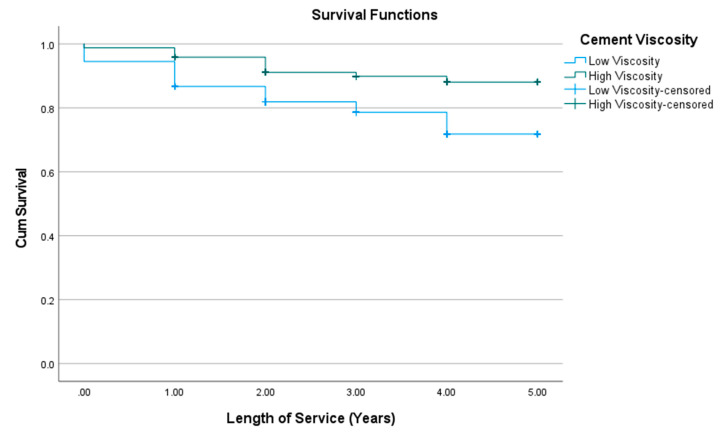
Kaplan-Meier survival curve of composite restorations stratified by cement viscosity, Palacky University Hospital, 2014–2018 (*n* = 469). CumSurvival = cumulative survival.

The cements with high viscosity had significantly (*χ*^2^ = 12.522; Sig. < 0.001) higher survival (4.64 ± 0.07) than those with low viscosity (4.14 ± 0.11) (Figure 6).

## 4. Discussion

### 4.1. Restoration Survival

A recent meta-analysis of Fan et al. 2021 revealed that survival rate of indirect composite inlays, onlays, and overlays after five years was 91% and the success rate was 84% [64]. Secondary caries and endodontic complications were predominant among composite onlays, and nonvital teeth and multiple-surface restoration were depicted as risk factors [64]. No direct connection was found between bruxism and fractures and no other factors influencing clinical outcome were found in this meta-analysis [64]. Another recent systematic review of Bustamante-Hernandez et al. 2020 concluded that ceramic onlays outperformed composite onlays based on 18 clinical trials, even though ceramic onlays were more prone to fracture and discoloration [65]

Survival and success rates of composite onlays were reported by several observational and experimental studies, e.g., Signore et al. 2007 found that indicated composite onlays, after 4–6 years of installation, exhibited a 93% survival rate, with a minority of patients requiring endodontic treatments [66]. Chrepa et al. 2014 evaluated 189 composite onlays received by 153 patients for 24 to 52 months, and they found that 2.1% of the onlays lost retention during the first week, and 1.1% broke up after 26–36 months [67]. While dual-cure self-etching resin cement TotalCem was used in this study, no data were found about gender or age associations with the clinical outcomes of these patients [67].

A randomized controlled trial (RCT) by Fennis et al. 2014 compared the direct versus the indirect composite restorations that provided coverage of the cusp for 5 years [68]. The investigators concluded that the differences in survival rates were not statistically significant in the premolar area where all the restorations were installed, and failure was attributed primarily to the adhesive [68]. Indirect restorations exhibited an 83.2% survival rate for both reparable and irreparable failures, and dislodgement was reported in 26.7% of the indirect restorations, and dislodgement and cohesive failure were reported in 20% [68]. It is worthy of note that dual-cured composite resin Panavia F was used in this RCT [68].

Dias et al. 2018 followed 150 endodontically treated molars and premolars, which received composite overlays [69]. The patients were recalled after two to five years; three reparable fractures (two males and one female) and two irreparable fractures (one male and one female) occurred during the follow-up [69]. There were no fractures of restored teeth nor debonding reported in this study, and all irreparable fractures occurred when the antagonist was an implant-supported ceramic crown [69]. It is worthy of mention that the adhesive cement Relyx Unicem was used in this study [69]. Kaytan et al. 2005 checked every 6 months for two years 94 ceramic onlays received by 47 patients [70]. No debonding, fracture, or discoloration was found in this study, which used dual-cured composite cement [70].

D’Arcangelo et al. 2014 observed 79 indirect composite restorations during five years, and found a 91% survival rate and 84.8% success rate after using preheated light-cured composite [71]. Two patients showed negative pulp vitality tests and four restorations were complicated by secondary caries [71]. One extensive restoration fracture was reported and two restorations lost adhesion between 36 and 48 months of service [71].

In our study, 84.9% of the onlays survived without the need for replacement or repair, and the highest percentage of failure came from 2014 and decreased over time. The restoration fracture caused 50% of our failure cases. These results are consistent with what had been reported earlier by Schulte et al. 2005 and Zimmer et al. 2008 [72,73]. However, Zimmer’s study was mainly on ceramic onlays and found a high success rate (84.9%) among ceramic onlays after 10 years of follow-up, the study also followed up to 95 patients with 388 composite onlays [73]. The most frequent complication was restoration loss (*n* = 10), followed by secondary caries that occurred exclusively during the first five years (*n* = 7), restoration fracture (*n* = 4), and tooth fracture (*n* = 2) [73]. Failure rate was found to be higher in the molar area compared to the premolar area, and the Cox regression analysis revealed no association between enamel cervical margin, bruxism, and prior root canal treatment. Moreover, it is worthy of note that only the Vita Cerec Duo cement was used in this study, which is a composite cement [73].

In our study, the higher survival rate was found for onlays compared with overlays; this can be explained in accordance with the systematic review and meta-analysis from Fan et al. 2021, which found that multiple surface restorations tend to have an increased risk of failure [64]. Malament et al. 2021 studied onlay survival for 10.9 years and did not find statistically significant difference depending on age. However, they found the lowest risk of failure for the group under 33 years of age [74]. The suggested hypothesis implies that, in younger age, the loss of hard dental tissue is limited compared to the older age, which can lead to indication of multiple surface restorations with increased risk of failure. This is just a possible explanation and our research cannot support this assumption.

### 4.2. Cement Viscosity

In the in vitro study of Hahn et al. 2001 for the impact of cement viscosity on microleakage, highly viscous cements had significantly better results on the cement/dentin interface [75]. Bortolotto et al. 2013 compared the behavior of composite resin versus resin cement in terms of shrinkage development and early solubility [76]. The lowest shrinkage was observed in composite resin cements, while the shrinkage development was slower in self-cure resin cements [76]. A recent in vitro study of Zeller et al. 2021 studied the viscosity and polymerization of three different composite resin-based cements; the investigators found that there were different reactions for each material upon preheating [77]. For Relyx Ultimate and Relyx Unicem, viscosity rises with preheating to 37 °C; however, the polymerization took place rapidly. These two cements, one adhesive (Relyx Ultimate) and another one self-adhesive (Relyx Unicem), were used in our study [77].

Mounajjed et al. 2018 found in an in vitro study that the marginal gap increased with preheated composite, thus suggesting that more precise placement is possible with highly viscous materials, as achieving smaller gaps can be impossible with preheated composite [42].

In contrast to our findings, Francescantonio et al. 2013 suggested that the smaller layer of cement and low-viscosity cements typically correlate with low polymerization stress and can effectively reduce cracks and premature edge penetrations [78]. Filtek Ultimate Flow, which was a material with low viscosity, failed multiple times in our study. Recently, Marcondes et al. 2020 compared the viscosity of flowable composite resin and resin-based luting cements, and found smaller viscosity in flowable composite [79]. Both groups of materials did not react in the same way upon preheating, as the viscosity of flowable composite was not lower after preheating. This fact for clinicians doubts the philosophy of preheating of composites, unless they do not require the material in detail [79].

Applying ultrasonic vibration by fixation of indirect composite restorations can be an alternative to using preheated composite. Cantoro et al. 2021 found, under electron microscopy, increased homogenous structure and reduced porosity in composite cements as a result of ultrasonic vibration. Nevertheless, this technique was not applied in our study [80].

Sato et al. 2014 suggested that rheology of the luting cements can be modified by adding 10-methacryloyloxydecyl dihydrogen phosphate (MDP), and, when high amounts of residual monomer remain in the cement, the crosslinking of the polymer web can be affected and the long-term stability can be jeopardized [81]. Given the complication of microleakage, the materials with higher viscosity can reduce it [75]. Recently, Zhang et al. 2021 found similar rheological proprieties in flowable resin composites and resin cements [82].

### 4.3. Polymerization

The abovementioned study of Francescantonio et al. 2013 found a higher degree of conversion for higher viscosity of cements activated with light [78]. The polymerization stress is significantly reduced in self-cure cements compared to the light-cured ones. Moreover, larger polymerization stress or inadequate polymerization in deeper cavities or under opaque restoration can lead to debonding [83].

Light curing and control above the setting is extremely important for clinicians; because of this reason, thiourethane additives can be used with resin cements. Thiourethane additives can reduce the polymerization stress and increase workability of dual-cured cements and the conversion rate can be increased. However, thiourethane additives affect optical proprieties of the material and reduce transparency [84,85,86,87].

### 4.4. Limitations

The first limitation of this study is related to the prosthetic restorations that were placed by different prosthodontists; therefore, the variability in survival and complications of the evaluated restorations could be partly attributed to the clinical/technical skills of the operators. The second limitation is due to the retrospective nature, which did not allow the investigators to assess subclinical characteristics of the participants. 

## 5. Conclusions

Indirect composite restoration offers a predictable and minimally invasive solution to hard dental tissue loss. The complications, survival, and success rates can be dependent on multiple factors, including the dentist, patient-related factors, material selection, and other factors. Within the limitations of our study, the composite indirect works present higher longevity when luted with high-viscosity cements. Moreover, the higher longevity was observed when the less invasive solution, e.g., onlays, was used. Based on our study, high-viscosity composite-based cements can be recommended for fixation of indirect composite restorations. During the indication of indirect work, it can be recommended to avoid cusp capping when it is not necessary. It can be recommended to pay attention to moisture control in the lower jaw, which is one possible reason for higher failure in the lower jaw.

## Figures and Tables

**Table 1:** Demographic and clinical characteristics of composite restoration recipients, Palacky University Hospital, 2014–2018 (*n* = 469).

Variable	Outcome	Frequency (*n*)	Percentage (*%*)
Sex	Female	271	57.8%
	Male	198	42.2%
Age	≤55 years old	354	75.5%
	>55 years old	112	23.9%
	Missed	3	0.6%
Quadrant	Upper Right	102	21.7%
	Upper Left	135	28.8%
	Lower Left	121	25.8%
	Lower Right	111	23.7%
Prosthetic Restoration	Onlay	372	79.3%
	Overlay	92	19.6%
	Inlay	5	1.1%
Cement Brand	Harvard Premium Flow	93	19.8%
	Enamel Plus	239	51%
	Filtek Ultimate Flow	47	10%
	Filtek Bulk Fill Flowable	6	1.3%
	Filtek Ultimate	7	1.5%
	Relyx Ultimate	65	13.9%
	Relyx Unicem	12	2.6%
Cement Material	Composite-based	392	83.6%
	Resin-based	77	16.4%
Cement Viscosity	Low Viscosity	223	47.5%
	High Viscosity	246	52.5%
Installation Year	2014	25	5.3%
	2015	105	22.4%
	2016	172	36.7%
	2017	148	31.6%
	2018	19	4.1%
Follow-up	Survived	398	84.9%
	Failed	71	15.1%
Failure Etiology	Leaked Filling	19	26.8%
	Fracture	36	50.7%
	Secondary Caries	16	22.5%

**Table 2:** Characteristics of survived vs. failed composite restorations, Palacky University Hospital, 2014–2018 (*n* = 469).

Variable	Outcome	Survival (*n* = 398)	Failure (*n* = 71)	Sig.
Sex	Female	229 (84.5%)	42 (15.5%)	0.799
	Male	169 (85.4%)	29 (14.6%)	
Age	≤55 years old	307 (86.7%)	47 (13.3%)	0.036
	>55 years old	88 (78.6%)	24 (21.4%)	
Arch	Upper Arch	207 (87.3%)	30 (12.7%)	0.125
	Lower Arch	190 (82.3%)	41 (17.7%)	
Prosthetic Restoration	Onlay	312 (83.9%)	60 (16.1%)	0.515 *
	Overlay	81 (88%)	11 (12%)	
	Inlay	5 (100%)	0 (0%)	
Cement Brand	Harvard Premium Flow	80 (86%)	13 (14%)	0.002 *
	Enamel Plus	215 (90%)	24 (10%)	
	Filtek Ultimate Flow	34 (72.3%)	13 (27.7%)	
	Filtek Bulk Fill Flowable	6 (100%)	0 (0%)	
	Filtek Ultimate	7 (100%)	0 (0%)	
	Relyx Ultimate	48 (73.8%)	17 (26.2%)	
	Relyx Unicem	8 (66.7%)	4 (33.3%)	
Cement Material	Composite-based	342 (87.2%)	50 (12.8%)	<0.001
	Resin-based	56 (72.7%)	21 (27.3%)	
Cement Viscosity	Low Viscosity	176 (78.9%)	47 (21.1%)	<0.001
	High Viscosity	222 (90.2%)	24 (9.8%)	
Installation Year	2014	15 (60%)	10 (40%)	<0.001 *
	2015	78 (74.3%)	27 (25.7%)	
	2016	149 (86.6%)	23 (13.4%)	
	2017	137 (92.6%)	11 (7.4%)	
	2018	19 (100%)	0 (0%)	

Chi-squared test (*χ*^2^) and Fisher’s exact test, * were used with a significance level (Sig.) of ≤ 0.05.

**Table 3:** Crude longevity of composite restorations, Palacky University Hospital, 2014–2018 (*n* = 469).

Variable	Outcome	Length of Service (Years)	Sig.
Sex	Female	2.59 ± 1.05	0.991
	Male	2.65 ± 1.12	
Age	≤55 years old	2.66 ± 1.06	0.255
	>55 years old	2.48 ± 1.13	
Arch	Upper Arch	2.67 ± 1.01	0.514
	Lower Arch	2.56 ± 1.15	
Prosthetic Restoration	Onlay	2.66 ± 1.09	0.024
	Overlay	2.43 ± 1.07	
	Inlay	2.60 ± 0.55	
Cement Brand	Harvard Premium Flow	2.20 ± 0.88	<0.001
	Enamel Plus	2.77 ± 0.97	
	Filtek Ultimate Flow	2.81 ± 1.15	
	Filtek Bulk Fill Flowable	2.00 ± 0.00	
	Filtek Ultimate	1.86 ± 0.38	
	Relyx Ultimate	2.68 ± 1.50	
	Relyx Unicem	2.55 ± 1.21	
Cement Material	Composite-based	2.61 ± 1.00	0.475
	Resin-based	2.66 ± 1.46	
Cement Viscosity	Low Viscosity	2.48 ± 1.18	0.011
	High Viscosity	2.74 ± 0.97	
Installation Year	2014	3.80 ± 1.73	<0.001
	2015	3.40 ± 1.19	
	2016	2.77 ± 0.67	
	2017	1.90 ± 0.38	
	2018	1.00 ± 0.00	

Mann-Whitney (U) test and Kruskal-Wallis (H) test have been used with a significance level (Sig.) of ≤ 0.05

**Table 4:** Regression analysis of composite restoration failure, Palacky University Hospital, 2014–2018 (*n* = 469).

Predictor	B (SE)	Wald (df)	AOR (CI 95%)	Sig.
>55 years old (vs. ≤55 years old)	0.52 (0.29)	3.36 (1)	1.69 (0.97–2.94)	0.067
Resin-based (vs. Composite-based)	1.06 (0.30)	11.98 (1)	2.90 (1.59–5.29)	<0.001
Low Viscosity (vs. High Viscosity)	0.94 (0.28)	11.72 (1)	2.57 (1.50–4.41)	<0.001

Logistic regression was executed with a significance level (Sig.) of ≤ 0.05.

**Table 5:** Equality of survival distributions for the different levels of failure risk factors, Palacky University Hospital, 2014–2018 (*n* = 469).

Log Rank (Mantel-Cox)	Chi-Squared	df	Sig.
Age Group	4.514	1	0.034
Cement Material	10.310	1	0.001
Cement Viscosity	12.522	1	<0.001

Kaplan-Meier analysis was executed with a significance level (Sig.) of ≤ 0.05

## Data Availability

The data that support the findings of this study are available from the corresponding author upon reasonable request.

## Supplementary Materials

The following are available online at https://www.mdpi.com/article/10.3390/ma15010312/s1, Table S1: STROBE Checklist of items that should be included in reports of cohort studies.docx.
